# Factors influencing nutritional practices among mothers in Dakar, Senegal

**DOI:** 10.1371/journal.pone.0211787

**Published:** 2019-02-11

**Authors:** Hye-Kyung Oh, Sunjoo Kang, Sung-Hyun Cho, Yeong-ju Ju, Daouda Faye

**Affiliations:** 1 College of Nursing, Seoul National University, Seoul, South Korea; 2 Department of Nursing, Gyeongbuk College of Health, Gimcheon-si, Gyeongsangbuk-do, South Korea; 3 Department of Nursing, Cheju Halla University, Jeju-si, Jeju Special Self-Governing Province, South Korea; 4 Department of Nursing, Chodang University, Muan County, Jeollanam-do, South Korea; 5 Department of Public Health, National School of Sanitary and Social Development, Dakar, Senegal; University of Texas Medical Branch at Galveston, UNITED STATES

## Abstract

**Background:**

Maternal undernutrition is a leading cause of maternal mortality. Furthermore, health statuses and habits of mothers influence health statuses of newborns as well as healthy habits and mortality of children. The Senegal government is aware of the severity of these issues and has devised a national policy goal of reducing maternal, infant, and adolescent mortality rates by the end of 2018. This study aimed to identify nutritional knowledge, attitudes, and practices of lactating women in Senegal, and determine factors related to nutritional practices to obtain basic data for developing a maternal and child nutrition project.

**Method:**

This study used a mixed–method approach, collecting data via structured questionnaires administered to lactating women in Senegal and semistructured interviews with seven stake–holders. Questions for stuctured questionnaires were about nutritional knowledge, attitudes, and practices. For the quantitative analysis of the structured questionnaires, data from 171 participants analyzed using independent t-tests, Pearson’s correlation coefficients, and multiple linear regression analysis. Interview data were analyzed using an inductive thematic analysis approach. The questions for the interviews concerned maternal and child nutritional status, causes of undernutrition, and restrictions.

**Results:**

Factors significantly related to healthy nutritional practices(explaining 27.1% of variance) included having a household (B = 1.03, *p* = .015) and a mother (B = 0.96, *p* = .017) with an above primary school education, and being in the 5th quintile of income level (B = 1.24, *p* = .014). The interviews with seven stakeholders revealed obstructive factors of nutritional management were insufficient nutritional programs within health centers, incomplete national policy on nutrition, lack of general interest in undernutrition-related topics, inadequate economic environment, and the absence of partnerships to produce sustainable solutions.

**Conclusion:**

Education and income levels, rather than knowledge and attitudes, had a strong relationship with healthy nutritional practices. Therefore, economic factors and educational background must be considered to succeed in Senegalese nutrition projects.

## Introduction

### Background

The United Nations (UN) has proposed good health and well-being for all as one of 17 sustainable development goals (SDGs) to facilitate global achievement by 2030 [1]. This pertains to a broader concept of health than has been used in the past, as reflection of the increased demand for health care in the international community. Despite the formulation of this goal, maternal and child health outcomes remain a major issue worldwide. Maternal and child undernutrition including stunting, wasting, and deficiencies of essential vitamins and minerals, represents a global problem with important results for survival, incidence of acute and chronic diseases, healthy development, and economic productivity [2–4]. Many women suffer from undernutrition in developing countries, but this issue has received little attention as an important determinant of poor maternal, newborn, and child health (MNCH) outcomes [5]. However, maternal undernutrition should not be overlooked because it contributes to deficits in children’s development and the health of child and mother [2, 4, 6, 7].

Approximately 830 women die daily from preventable causes during pregnancy and childbirth; nearly 99% of maternal deaths from theses causes occur in underdeveloped countries [8]. Iron and calcium deficiencies substantially affect maternal mortality and mothers presenting severe anemia are more likely to die from hemorrhage during childbirth [9]. Maternal mortality is one of the main indicators of maternal health and is measured globally as the number of maternal deaths per 100,000 births. The current global maternal mortality rate is 216, with the highest rate being in sub–Saharan Africa; there, the average rate is 2.5 times that of the global average, at nearly 546 deaths [8, 9]. Among these sub–Sahara African countries, Senegal had a maternal mortality of 320 deaths per 100,000 births in 2013. While substantially below the average within sub–Saharan Africa, it remains significantly higher than the global average of 216. Senegal has shown significant progress in achieving several of the Millennium Development Goals (MDGs), including universal primary education (Goal 2), gender equality (Goal 3), reduction in the incidence rate of HIV/AIDS and malaria (Goal 6), and sustainable environment protection (Goal 7); however, reduction in the maternal and child mortality rate has progressed at a much slower rate, such that it fell short of the MDG target in 2015 [10–12]. A possible reason for the slower progression is the high prevalence undernutrition and anemia, which are associated with mortality in Senegal [13]. In 2016, 49.9% of Senegalese women of reproductive age suffered from anemia, while the prevalence of anemia among Senegalese pregnant women was 58.1% [14]. The nutritional status of reproductive women is one of the determinants of maternal mortality and uneventful pregnancies; however, in 2011 a relatively high ratio (22%) of Senegalese women were below the critical body mass index (BMI) threshold of 18.5 [13]. Many studies have shown that BMI is preferred as an indicator of malnutrition [15–17], as it corresponds to a general lack of energy and nutrition.

In 2015, the global mortality rate for children under the age of 5 was 43 per 1000 population. Again, the average rate for Africa far outstripped the global average by 1.9 times, at 81.3; this was the highest rate worldwide [18, 19]. In 2015, the under–5 mortality rate was 47.2 per 1000 population in Senegal [20]. In 2012–2013, the prevalence of stunted growth among Senegalese children under 5 years of age was 19%, of which 6% had severe stunting. Furthermore, the prevalence of anemia among Senegalese children under 5 years of age was 71%, the majority of whom had moderate anemia. Undernutrition is a result of scarce resources and education, which are major causes of child mortality in underdeveloped countries [21].

Undernutrition in the mother has direct effects on maternal mortality and children’s healthy development and health status. It is especially important to ensure that lactating women maintain a good nutritional status, as it is directly linked to the developing child’s health. Therefore, this study was conducted to identify the actual state of nutritional knowledge, attitudes, and practice among Senegalese lactating women, and to identify the factors that influence nutritional practice, using questionnaires and interviews. The general aim was to identify the factors affecting nutritional practice for developing and implementing a local nutrition project. Taking these factors into account before beginning a nutritional project may help to minimize the risk of failure.

## Materials and methods

### Study design

The study adopted a mixed method approach to examine the influencing factors of nutritional practices in Senegal. The quantitative study involved administering structured questionnaires to lactating women, while the qualitative study involved conducting semistructured interviews face-to-face with seven stakeholders.

### Participants

The study participants were lactating women visiting two health centers selected randomly who expressed a desire to voluntarily participate in the study. The questionnaire surveys were conducted in December 2017. The questionnaire was written in French and was administered by two local researchers fluent in French, English, and the local dialects. For the quantitative study, the sample size was calculated using G*power 3.0.10 based on a significance level of 0.05, a medium effect size (0.15), a power of 0.95, and 12 independent variables. According to this analysis, the minimum sample size was 171, although 184 were ultimately recruited with consideration of the dropout rate. After the completed questionnaires were received, a preprocessing step was conducted to remove incomplete or invalid data and non-target subjects. The final sample size was 171 subjects. For the qualitative study, five nursing professors from South Korea prepared a list of relevant stake–holders through a meeting. The interview participants included two maternal and child health officers of the Ministry of Health, two directors of health centers, a professor of a health university, and two lactating women. The official letters for interviews were processed through the Korea International Cooperation Agency (KOICA) Senegal office. The semistructured interviews were conducted from December 1 to 8, 2017. The interview site was conference rooms or health centers of each institution.

### Research ethics

This study was approved by the bioethics committee of a public institution affiliated with the Ministry of Health and Welfare, Republic of Korea (P01–201712–22–001). Documents containing explanations of the purpose, methods, and procedure of the study; the confidentiality and anonymity of the data; and the fact that the subjects could stop participation at any time for any reasons were distributed. Subsequently, all participants’ informed written consent to participate in the study was obtained.

### Variables and instruments

The nine items concerning participants’ personal characteristics were constructed by a researcher based on previous studies, and focused on age, infants’ average gestational age in months, the number of children in the household, participants’ marital status, the head of household and that person’s education level, participants’ education level, the number of incomes–generating activities in the family, and the family’s wealth index. The guidelines for assessing nutrition–related knowledge, attitudes, and practices provided by the Food and Agriculture Organization of the United Nations were used to assess participants’ nutrition–and health–related knowledge, attitudes, and practices [22]. Nutritional knowledge consists of women’s nutrition during pregnancy and breastfeeding, micronutrient supplements for pregnant women, recommendation of folic acid supplements, health risks for low–birth–weight babies, and family planning. There 5-items measuring nutritional knowledge were rated on a dichotomous scale (0 = “does not know” response, 1 = “knows”). For each question, a correct response was coded as 1 and an incorrect response as 0. The total score was calculated from all the correct responses, with a maximum of 5. Nutritional attitudes consisted of attitudes toward health and nutritional problems and eating more food during pregnancy. They were measured with a 4-item questionnaire related to perceived susceptibility, severity, benefits, and barriers; each item was rated on a 3-point Likert scale (1 = “not likely/serious/good/difficult”, 2 = “you are not sure/so-so’, 3 = ‘likely/serious/good/difficult”). For each item, the response score was reflected in the result value. Nutritional practice involved checking dietary diversity, based on what participants had eaten in the previous 24 hours. Foods were categorized into 9 groups: starchy foods, legumes/nuts, milk/mild products, organ meat, flesh meat/fish, eggs, other fruits/vegetables, dark green leafy vegetables, and fruits/vegetables rich in vitamin A. The total frequency of consumption of these categories of foods was taken as the nutritional practice scores, with a maximum of 9.

The 2 interview questions related to maternal and child nutritional status, causes of undernutrition, and restrictions of activities to improve the nutrition status of mothers and children.

### Data analysis

All collected data were analyzed using SPSS Statistics 22.0 (IBM Corp., Armonk, MY, USA). We described participants’ demographic characteristics, nutritional knowledge, attitudes, and practices using real numbers, percentages, means, and standard deviations. We used the Cronbach’s α coefficient to verify the internal reliability of the measures. We analyzed differences in the participants’ nutritional knowledge, attitudes, and practices according to participants’ general characteristics using independent t-tests. The correlations among nutritional knowledge, attitudes, and practices were analyzed using Pearson’s correlation coefficients. Finally, the influencing factors of participants’ nutritional practices were analyzed using multiple linear regression analyses. The dependent variable in these analyses was nutritional practice, and the independent variables were the general characteristics and nutritional knowledge and attitude. The interview data were analyzed using an inductive thematic analysis approach for the thematic content analysis.

## Results

### General characteristics

Participants of this study ranged in age from the high teens to mid-forties. The average gestational age in months was 9 months, most of the participants were married (94.7%). Regarding the household head, most participants came from household headed by males (84.2%), while 14.0% came from households headed by females. As for the education levels of participants’ household heads, 58.5% of household heads had not completed primary school, 15.8% had completed primary school, and 21.1% had an above primary school education. Among the education levels of the participants, the distribution was similar to that of household heads: 48.0% had not completed primary school graduation, 22.2% had completed primary school, and 24.5% had above a primary school education. As for income–generating activities, almost all participants came from a household with no or one income–generating activity (93.5%). Among the income levels, the lowest proportion of participants (11.7%) was in the 1^st^ quintile, while the highest proportion (31.6%) was in the 5^th^ quintile (see Table 1).

**Table 1:** Demographic characteristics of subjects (N = 171).

Characteristics	Classification	Frequency (%)	M±SD
Age (years)	18–20	50(29.2)	26.40±7.64
	21–25	37(21.7)	
	26–30	36(21.1)	
	31–35	19(11.1)	
	36–40	19(11.1)	
	Over 40	10(5.8)	
Average gestational age (months)	Under 9 months	1(0.5)	
	9 months	158(92.4)	
	No answer	12(7.1)	
Number of children	1	43(25.2)	
	2	37(21.6)	
	3	37(21.6)	
	4	27(15.8)	
	5 or more	27(15.8)	
Marital status	Single	8(4.7)	
	Married	162(94.7)	
	Divorced/separated	1(0.6)	
Household head	Male household head	144(84.2)	
	Female household head with male support	14(8.2)	
	Female household head without male support	10(5.8)	
	No answer	3(1.8)	
Household head education level	Did not complete primary school	100(58.5)	
	Primary school completed	27(15.8)	
	Above primary school	36(21.1)	
	No answer	8(4.7)	
Mothers’ education level	Did not complete primary school	82(48.0)	
	Primary school completed	38(22.2)	
	Above primary school	42(24.5)	
	No answer	9(5.3)	
Income–generating activities	None	83(48.5)	
	One activity	77(45.0)	
	Two activities	3(1.8)	
	Three activities	1(0.6)	
	No answer	7(4.1)	
Wealth index, quintile	1^st^ quintile (lowest)	20(11.7)	
	2^nd^ quintile	4(2.3)	
	3^rd^ quintile	70(40.9)	
	4^th^ quintile	23(13.5)	
	5^th^ quintile (highest)	54(31.6)	

### Nutritional knowledge, attitudes, and practices

The mean nutritional knowledge score for lactating women was 2.41±2.15 (mean±SD) out of a total of 5. The mean scores of nutritional attitudes were 2.78±0.49, 1.49±0.74, 2.01±0.45, and 2.78±0.47 (out of a total of 3) for perceived benefits, perceived barriers, perceived susceptibility, and perceived severity, respectively. The mean score for nutritional practices, which reflected the degree of dietary diversity, was 4.41±1.97 out of a total of 9 (see Table 2).

**Table 2:** Knowledge, attitudes and practices of nutrition (N = 171).

Variable	M±SD	Min	Max	Cronbach’s
Nutritional knowledge score	2.41±2.15	0	5	0.914
Nutritional attitude score				
Perceived benefits	2.78±0.49	1	3	
Perceived barriers	1.49±0.74	1	3	
Perceived susceptibility	2.01±0.45	1	3	
Perceived severity	2.78±0.47	1	3	
Nutritional practice score	4.41±1.97	0	9	0.646
Starchy foods	0.97±0.18	0	1	
Legumes and nuts	0.38±0.49	0	1	
Milk and milk products	0.43±0.50	0	1	
Organ meat	0.17±0.38	0	1	
Flesh meat and fish	0.88±0.32	0	1	
Eggs	0.46±0.50	0	1	
Other fruits and vegetables	0.40±0.49	0	1	
Dark green leafy vegetables	0.16±0.37	0	1	
Fruits and vegetables rich in Vitamin A	0.52±0.50	0	1	

### Differences in nutritional knowledge, attitudes, and practices according to general characteristics

We found significant differences in nutritional knowledge scores according to whether participants lived with a spouse or not (t = -2.82, *p =* .017), participants’ education levels (t = 3.12, *p =* .047), and income levels (t = 11.41, *p* < .001). An ex-post analysis revealed that, for the education levels, participants who had not completed primary school had higher nutritional knowledge scores than did those who had an above primary school graduation. Among income levels, participants in the 1^st^ and 2^nd^ quintiles had higher nutritional knowledge scores than did those in the 3^rd^ and 5^th^ quintiles. We also found differences in perceived susceptibility (an attitude measure) according to whether participants were living with the spouse or not (t = 2.59, *p =* .030). Nutritional practice scores significantly differed according to whether participants were living with the spouse or not (t = 2.54, *p* = .012), household head (t = 7.55, *p* = .001), household head’s education level (t = 18.07, *p* < .001), participants’ education levels (t = 14.01, *p* < .001), and income levels (t = 11.84, *p* < .001). The results of an ex-post analysis revealed that participants from a household with a female head (both supported and not supported by males) had higher nutritional practice scores than did participants from a household with a male head. As for household head’s education level, participants whose household head had an above primary school graduation had higher scores than did those who had not completed primary school.

Participants who did not complete primary school had lower nutritional practices scores than did those who had completed primary school. Finally, participants in the 5^th^ income quintile showed significantly higher practice scores than did those in the 1^st^, 2^nd^, and 3^rd^ quintiles (see Table 3).

**Table 3:** Nutritional knowledge, attitudes, and practices according to general characteristics.

Characteristics	Knowledge			Attitude												Practice		
				Perceived susceptibility			Perceived severity			Perceived benefits			Perceived barriers					
	M±SD	t/F	*p*	M±SD	t/F	*p*	M±SD	t/F	*p*	M±SD	t/F	*p*	M±SD	t/F	*p*	M±SD	t/F	*p*
Age																		
Under 26 years	2.19±2.17	-1.38	.168	2.00±0.38	-.18	.861	2.79±0.46	.51	.612	2.82±0.42	1.16	.248	1.49±0.76	.01	.996	4.59±1.89	1.27	.206
27 years or older	2.64±2.12			2.01±0.51			2.76±0.49			2.73±0.57			1.49±0.73			4.21±2.05		
Number of children																		
2 or fewer	2.19±2.08	-.52	.606	1.99±0.47	-.70	.486	2.76±0.49	-.57	.571	2.76±0.51	.25	.807	1.48±0.73	.15	.881	4.74±2.03	1.73	.086
3 or more	2.41±2.20			2.06±0.54			2.81±0.46			2.73±0.61			1.46±0.69			4.06±1.82		
Living with the spouse or not																		
No	1.11±1.36	-2.82	.017	2.44±0.53	2.59	.030	2.89±0.33	.74	.459	2.67±0.71	-.67	.502	1.67±0.87	.72	.474	6.00±1.87	2.54	.012
Yes	2.48±2.17			1.98±0.43			2.77±0.48			2.78±0.49			1.48±0.73			4.31±1.94		
Form of household head																		
Male household head^a^	2.49±2.19	.19	.826	2.02±0.41	2.14	.121	2.79±0.44	.62	.540	2.82±0.42	1.70	.187	1.51±0.76	.17	.847	4.13±1.89	7.55	.001 (a<b,c)*
Female household head with male support^b^	2.29±1.94			1.79±0.58			2.64±0.75			2.64±0.75			1.43±0.51			5.64±1.78		
Female household head without male support^c^	2.10±1.97			1.90±0.57			2.80±0.42			2.60±0.70			1.40±0.84			1.97±0.62		
Household head’s education level																		
Did not complete primary school^a^	2.61±2.26	1.52	.223	2.01±0.34	.42	.661	2.85±0.36	2.66	.073	2.82±0.44	.99	.373	1.56±0.77	1.09	.338	3.77±1.63	18.07	< .001 (a,b<c)*
Primary school completed^b^	2.56±2.14			1.92±0.64			2.63±0.63			2.74±0.60			1.52±0.80			4.63±2.19		
Above primary school^c^	1.89±1.88			2.00±0.54			2.75±0.50			2.69±0.58			1.34±0.64			5.86±1.90		
Maternal education level																		
Did not complete primary school^a^	2.84±2.25	3.12	.047 (a>c)*	2.00±0.23	.20	.818	2.79±0.41	.07	.933	2.79±0.44	.35	.704	1.50±0.72	.10	.904	3.67±1.59	14.01	< .001 (a<b,c)*
Primary school completed^b^	2.55±2.26			2.00±0.62			2.76±0.55			2.78±0.53			1.47±0.80			4.66±1.77		
Above primary school^c^	1.83±1.72			1.95±0.50			2.79±0.47			2.71±0.60			1.55±0.77			5.48±2.23		
Income-generating activities																		
None	2.45±2.12	-.14	.887	2.00±0.35	.37	.715	2.81±0.42	1.16	.250	2.80±0.46	.85	.395	1.48±0.72	-.16	.875	4.43±1.89	.06	.950
At least one	2.49±2.19			1.97±0.51			2.73±0.53			2.74±0.55			1.50±0.75			4.41±2.08		
Income levels																		
1 or 2^a^	3.83±1.88	11.41	< .001 (a>b,d)*	2.00±0.00	.14	.936	2.92±0.28	.96	.415	2.88±0.45	.39	.758	1.58±0.83	.57	.637	3.33±1.40	11.84	< .001 (d>a,b)*
3^b^	1.63±2.12			1.99±0.37			2.76±0.46			2.77±0.46			1.54±0.72			3.85±1.79		
4^c^	3.74±1.76			2.00±0.30			2.78±0.42			2.74±0.54			1.35±0.71			4.52±1.31		
5^d^	2.22±1.91			2.04±0.65			2.72±0.56			2.75±0.55			1.45±0.75			5.52±2.12		

Note: P-values are in parentheses.

*p < .05 in Scheffé’s test

### Relationships between nutritional knowledge, attitudes, and practices

The correlations between the major variables are shown in Table 4. Nutritional knowledge showed a significant positive correlation with perceived benefits (r = 0.18, *p* = .019) and a significant negative correlation with perceived barriers (r = -0.28, *p <* .001). Perceived susceptibility showed a significant positive correlation with perceived severity (r = 0.18, *p* = .021), while perceived severity showed a positive correlation with perceived benefits (r = 0.49, *p* < .001). Perceived benefits had a negative correlation with perceived barriers (r = -0.16, *p =* .042) and perceived barriers had a negative correlation with nutritional practices (r = -0.18, p = .022).

**Table 4:** Correlations between nutritional knowledge, attitudes, and practices.

	Knowledge	Attitude				Practice
		Susceptibility	Severity	Benefits	Barriers	
Knowledge	1					
Susceptibility	-0.12(.135)	1				
Severity	0.14(.072)	0.18(.021) *	1			
Benefits	0.18(.019) *	0.06(.479)	0.49 (< .001) *	1		
Barriers	-0.28 (< .001) *	0.02(.811)	-0.03(.710)	-0.16(.042) *	1	
Practice	-0.02(.816)	-0.02(.833)	-0.10(.194)	-0.08(.328)	-0.18(.022) *	1

Note:

*p < .05

### Factors influencing nutritional practices

Multiple regression analyses were conducted to identify the factors that independently related to nutritional practices. The variance inflation factors (VIFs) and tolerances values were checked to check for multicollinearity. However, there was no problem of multicollinearity because the VIFs were 1.163–3.118 (and therefore were, smaller than the reference value of 10), and the range of tolerance values was 0.323–0.896 (and thus were larger than 0.1 but did not exceed 10). The independence of the residuals was checked with the Durbin–Watson statistic; as the statistic was 1.421, there was no problem of autocorrelation. The Cook’s distance for outliers was 0.101 or smaller, and thus were all smaller than the reference value of 1.0, therefore, the assumptions for the multiple regression analyses were satisfied.

Table 5 shows the results of the regression analyses, which was conducted with a total of 10 predictor variables: age, the number of children, living with a spouse or not, household head, household head’s education level, participants’ education level, number of income–generating activities, income level, nutritional knowledge, and nutritional attitudes. The regression model was found to be significant (F = 4.35, *p* < .001), and the adjusted coefficient of determination (Adj R²), which indicates the explanatory power of the model, was 0.27. The significant factors related to nutritional practice included having a household (B = 1.03, *p* = .015) a mother (B = 0.96, *p* = .017) with an above primary school education, and being in the 5^th^ income quintile (B = 1.24, *p* = .014). These together explained 27.1% of the variance in nutritional practice.

**Table 5:** Factors influencing nutritional practices.

Variables	Category	B	SE	β	t	*p*
Constant		5.19	1.71		3.04	.003
Age	(Score)	0.02	0.02	0.06	-.77	.442
Number of children	(Score)	0.05	0.09	0.05	0.54	.589
Living with spouse or not	Yes	0.59	0.73	0.07	0.80	.423
Household head	Male household head	0				
	Female household head with male support	0.88	0.51	0.13	1.72	.087
	Female household head without male support	0.41	0.70	0.05	0.59	.554
Household head’s education level	Did not complete primary school	0				
	Primary school completed	0.21	0.42	0.04	0.52	.607
	Above primary school	1.03	0.42	0.22	2.47	.015
Maternal education level	Did not complete primary school	0				
	Primary school completed	0.55	0.35	0.12	1.59	.115
	Above primary school	0.96	0.39	0.21	2.43	.017
Income generating activities	Yes	0.22	0.29	0.06	0.77	.444
Quintile	1 or 2	0				
	3	0.19	0.45	0.05	0.41	.684
	4	0.73	0.51	0.13	1.44	.152
	5	1.24	0.50	0.30	2.49	.014
Knowledge	(Score)	0.02	0.08	0.02	0.26	.792
Susceptibility	(Score)	0.13	0.33	0.03	0.41	.685
Severity	(Score)	0.22	0.34	0.05	0.66	.511
Benefit	(Score)	0.05	0.32	0.01	0.15	.880
Barrier	(Score)	0.45	0.20	0.17	2.31	.023
F(*p)*	4.35 (< .001)					
Adjusted R²	.271					

### Content analysis of semistructured interviews

Regarding the results of the analysis of the semistructured interviews with stake–holders, all stake–holders answered that the maternal and child nutritional status was a serious issue. The causes of undernutrition and restrictions were classified into five obstructive factors regarding maternal and child nutritional management: insufficient nutritional programs, incomplete national policy on nutrition, lack of general interest on undernutrition-related topics, inadequate economic environment, and the absence of partnerships to produce sustainable solutions (see Table 6).

**Table 6:** Content analysis of semistructured interviews with stake–holders.

Themes	Sub-themes	Statements (N)	N (%)
Maternal and child nutritional status	At a serious level	In rural areas, undernutrition is a major cause of child mortality (6)	7 (29.2)
		45% of child mortality is undernutrition (1)	
Causes of undernutrition and restrictions	Insufficient nutritional programs	There is no regular nutrition education program at the health center (2)	4 (16.6)
		There is no routine hemoglobin test for mothers and children (2)	
	Incomplete national policy on nutrition	There is no systematic management and policy of undernutrition (3)	3 (12.5)
	Lack of general interest on undernutrition	Most people are unaware of the seriousness of undernutrition (2)	3 (12.5)
		It is not an urgent disease that tends to fall behind in priorities (1)	
	Inadequate economic environment	The biggest cause of undernutrition is economic reasons (5)	5 (20.8)
	The absence of partnerships to produce sustainable solutions	Until now, there were many fragmented health projects only when funds were received from outside (1)	1 (4.2)
		There was a difference between the priorities of health problems in our country and those of other countries or institutions (1)	1 (4.2)
2	6	Total	24 (100.0)

## Discussions

In the present study, when we converted participants’ nutritional knowledge scores into categories, we found that 45.0% of participants had low knowledge scores, 11.7% had medium scores, and 43.3% had high knowledge scores. These results differ from those obtained by Perumal et al., [23] with pregnant women in Kenya, East Africa, which indicated that 33.9% of the participants had low knowledge scores, 44.6% had medium scores, and 21.5% had high knowledge scores; similar percentages were found for Kenyan youths [24]. By contrast, in this study, a much larger percentage of lactating women had high knowledge scores than medium knowledge scores. The observed difference might be due to differences in study participants’ level of education, exposure to nutritional education, and regional selection. The results for nutritional attitude in the study with the majority of the subjects having medium or high attitude scores were similar to those of many studies conducted in developing countries [21, 23, 25]. In this study, most participants had medium or low practice scores (85.9%), unlike in Perumal et al. [23], where 85.7% of participants reported medium practice scores. Low practice scores appear to directly reflect the poor nutrition status of lactating women, which is highly prevalent in Senegal due to the intergenerational cycle of undernutrition and poverty [26]. The issue here is not just the high nutritional knowledge and attitude scores, but how much these scores affect participants’ nutritional practice scores. Many studies have shown conflicting results as to whether nutritional knowledge and attitude actually affect practice. Okunaiya et al. [25] showed that adequate nutritional knowledge and attitude influence practice. Masuku and Lan [27], on the other hand, found that the nutritional knowledge and attitude of pregnant and lactating women in Swaziland did not necessarily translate into nutritional practice. Kigaru et al. [24], in a study of youths, found that nutritional knowledge did not affect nutritional practice, but that attitude was an important influencing factor of practice. However, it is difficult to compare their results to that of this study, given that they measured attitude simply in terms of caring about eating or not. Therefore, the factors affecting nutritional practice should be analyzed with great consideration of the the regional and target population characteristics and the contents of the instrument being used.

In analyzing the differences in nutritional knowledge, attitudes, and practices according to general characteristics, we found that participants with education levels lower than primary school tended to have higher nutritional knowledge scores than did those with high education levels, as did participants in the 1^st^ and 2^nd^ income quintiles (vs. those in the 3^rd^ and 5^th^ quintiles). Although these results cannot be easily interpreted because there are no previous studies on this topic, they can be attributed to the fact that nutrition education tends to focus on low-income individuals through public institutions such as health centers [28]. This indicates that academic background and certain areas’ of knowledge are not always proportional.

We found that the household head’s and participants’ own education levels were positively correlated with nutritional practices, and that participants in the 5^th^ income quintile had significantly higher practice scores than did those in the 1^st^, 2^nd^, and 3^rd^ quintiles. Previous research indicated that this is because women with high economic statuses and education levels are more likely to have girls that end up receiving a good education, which is the most effective method of reducing poverty and undernutrition [27, 29]. Nutritional and anemia status of children differ 2–3 times depending on the educational level of mother and wealth quintile [13]. That is, income and education level are both directly related to a decrease in the cycle of poverty.

The factors that influence nutritional practice included having an above primary school education and being in the 5^th^ income quintile. These findings are consistent with previous studies [25, 27, 30] showing that nutritional knowledge and attitudes are not directly related to practice, whereas, economic barriers and education levels are strong influencing factors. Ambadekar and Zodpey [31] and Lloyd [21] emphasized that economic status is an important factor in determining undernutrition. Therefore, future nutritional projects should include components centered on promoting community-based economic activities to enhance the economic independence of participants.

The obstructive factors to improving maternal and child nutrition status are insufficient nutritional programs, incomplete national policy on nutrition, lack of general interest in undernutrition, inadequate economic environment, and the absence of partnerships to produce sustainable solutions. To overcome these obstacles, it is necessary to strengthen public relations on nutrition issues, as well as to develop a nutrition education program that considers economic factors and builds partnership between families, communities and the government. Since encouraging nutritional practices can more directly impact maternal nutrition status compared to enhancing nutritional knowledge, our identification of the related factors of nutritional practices is meaningful for the implementation of nutrition projects in the future.

## Conclusions

The critical results obtained in this study are as follows:

First, education and income levels appear to have stronger effects on nutritional practice than nutritional knowledge and attitudes.

Second, it is important to identify the obstructive factors to improving maternal and child nutrition.

However, this study also has some limitations that must be addressed in future studies.

First, the participants were recruited through convenience sampling only in the urban areas of Senegal. Although this sampling is meaningful because nutrition deficiency is quite prevalent in Dakar, Senegal, due to rapid urbanization and increasing food prices [32], in future studies, it is necessary to check for regional differences in results by including mothers residing in urban and rural areas.

Second, our findings are subject to the limitations inherent in cross–sectional data. Therefore, in future studies, the causal relationships among variables should be analyzed through longitudinal study designs.

## Supporting information

S1 File Questionnaire (French). (DOCX)

S2 File Questionnaire (English). (DOCX)
