# Supplementary material for: Factors influencing nutritional practices among mothers in Dakar, Senegal
Source: PLoS One. 2019 Feb 11;14(2):e0211787. doi: 10.1371/journal.pone.0211787 (PMC6370274; doi:10.1371/journal.pone.0211787)
Supplement: S1 File — (DOCX) [file pone.0211787.s001.docx]

**Informations concernant le contenu et la confidentialité du questionnaire**

Bonjour/Bonsoir, Monsieur/Madame ________. Nous sommes membres de l'École de Santé de

Gyeongbuk en Corée du Sud. Nous effectuons un sondage au sein des participants afin d'évaluer leurs

savoir-faire, attitudes et pratiques concernant la nutrition. Ce questionnaire durera en moyenne

30minutes. Toutes les informations que vous nous communiquerez resteront strictement confidentielles

et vos réponses et informations personnelles ne seront en aucun cas révélées. De plus, vous n'êtes en

aucun cas tenu(e) de répondre à ce questionnaire si vous ne le souhaitez pas, et vous pouvez cesser de

répondre à tout moment.

L'objectif de cette étude est d'effectuer un sondage à propos du savoir-faire concernant la santé et la

nutrition, les attitudes et pratiques des mères de famille. Le but est en aucun cas de vous évaluer ou de

vous critiquer, de ce fait, ne vous sentez pas contraint(e) de donner une réponse spécifique et ne soyez

pas intimidé(e) si vous ne connaissez pas la réponse à une question. Nous ne nous attendons pas à ce que

vous donniez une réponse spécifique; Nous espérons que vous nous donnerez une réponse honnête, tout

en partageant avec nous ce que vous savez, comment vous vous sentez, votre train de vie et vos

habitudes culinaires. Prenez autant de temps nécessaire pour répondre à ce questionnaire.

Souhaitez-vous prendre part à ce questionnaire ?

Oui ___Non ___*Si Oui, vous pouvez procéder à la question suivante ; si Non, vous pouvez mettre fin au*

*questionnaire*.

Avant de débuter, avez-vous des questions ? (*Répondre aux questions*).

Êtes-vous prêt(e) à commencer ?

Décembre 2017.

Oh Hye-Kyung, Ju Young Ju, Lee Se Hoon, Kang Sun joo

E-mail : katie5@gch.ac.kr

Questionnaire Socio-démographique

1. Âge : ( )

2. Âge de gestation moyenne (mois) : ( )

3. Nombre d'enfants: ( )

Date de naissance du premier enfant: ( année, mois)

deuxième enfant: ( année, mois)

troisième enfant: ( année, mois)

quatrième enfant: ( année, mois)

4. État Civil

① Célibataire ② Marié(e) ③ Divorcé(e)/séparé(e)

**5.** Chef de famille

① Homme ② Femme avec soutient masculin ③ Femme sans soutient masculin

6. Niveau d'éducation du chef de famille

① Inférieur au niveau élémentaire ② École primaire complétée ③ Supérieur au niveau primaire

7. Niveau d'éducation de la mère

① Inférieur au niveau primaire ② École primaire complétée ③ Supérieur au niveau primaire

8. Activitées générant un revenu

① Pas impliqué(e) ② Impliqué(e) dans une activité ③ Impliqué(e) dans deux activités

④ Impliqué(e) dans trois activités

9. Possédez-vous une télévision au sein de votre domicile? □ Oui □ Non

10. Possédez-vous un réfrigérateur au sein de votre domicile? □ Oui □ Non

11. Possédez-vous une antenne MMDS/TV5 au sein de votre domicile? □ Oui □ Non

12. Possédez-vous un téléphone fixe au sein de votre domicile? □ Oui □ Non

13. Avez-vous accès à l’électricité au sein de votre domicile? □ Oui □ Non

14. Possédez-vous un lecteur CD/DVD au sein de votre domicile? □ Oui □ Non

15. Possédez-vous une connexion internet au sein de votre domicile? □ Oui □ Non

16. Possédez-vous un abonnement CANAL au sein de votre domicile? □ Oui □ Non

17. Possédez-vous un ordinateur au sein de votre domicile? □ Oui □ Non

18. Est-ce que certains membres de votre famille possèdent leur propre véhicule?

□ Oui □ Non

19. Est-ce que certains membres de votre famille possèdent leur propre carte de

crédit ? □ Oui □ Non

20. Est-ce que certains membres de votre famille possèdent leur propre compte

bancaire/ compte au sein d’un institution financière ? □ Oui □ Non

21. Quel type de feu utilisez-vous au sein de votre domicile pour cuisiner ?

① Bouteille de gaz ② Bois ③ Autre( )

22. Quelle est la principale source d’hydratation au sein de votre domicile ?

① Eau courante ② Puits non protégé ③ Autre( )

23. Quel type d’infrastructure sanitaire possédez-vous au sein de votre domicile ?

① Système d’évacuation des eaux ② Fosse septique ③ Toilettes

traditionnelles/rustiques ④ Autre( )

24. Quel est le matériau principal utilisé pour le sol de votre logement?

① Ciment ② carreaux ③ terre/sable ④Autre( )

25. Quel est le matériau principal utilisé pour le toit de votre logement?

① Ciment ② Calamine/fibre de ciment ③ Autre( )

26. Quel est le matériau principal utilisé pour les murs extérieurs de votre logement?

① Ciment ② Bambou/Cane/Palmier/Troncs/Terre ③ Autre( )

| *Groupe* | **Liste d'aliments** | **Non** | **Oui** |
| --- | --- | --- | --- |
| ***Groupe 1****:*  *Graines,*  *racines et*  *tubercules* | Porridge ,pain, riz, nouilles ou autre aliment à base de  grains |  |  |
|  | Pommes de terre, patates douces, manioc ou autre  aliment à base de racines |  |  |
| ***Groupe 2****:*  *Légumes et*  *noix* | Tout aliment à base d'haricot, petit pois, lentille, noix  ou graines |  |  |
| ***Groupe 3****:*  *Produits*  *laitiers* | Lait concentré, en poudre ou fraîchement traie  Yaourt ou lait caillé |  | Nombre de  fois?  \|___\|\|___\|  Nombre de  fois?  \|___\|\|___\| |
|  | Fromage ou autres produits laitiers |  |  |
| ***Groupe 4****:*  *Aliments de*  *chair* | Foie, rein, cœur ou autres organes d'animaux |  |  |
|  | Toute sorte de viande, par exemple bœuf, porc,  mouton, chèvre, poulet ou canard |  |  |
|  | Poisson frais ou séché, crustacés ou fruits de mer |  |  |
|  | asticots, escargots or insectes |  |  |
| ***Groupe 5****:*  *Œufs* | Œufs |  |  |
| ***Groupe 6****:*  *Fruits et*  *légumes* | Citrouille, carottes, courges ou patates douces de  couleur jaune ou orange à l'intérieur |  |  |
|  | Tout légumes verts |  |  |
|  | Mangues mûres (fraîches ou séchées), papayes mûres  (fraîches ou séchées), melon |  |  |

**Nutrition durant la grossesse et l'allaitement**

Nous allons maintenant vous posez quelques questions concernant l'alimentation des femmes en état de

grossesse ou qui allaitent.

**Q) P.1:** Nous allons maintenant vous posez quelques questions concernant liquides ou aliments que

vous avez consommé durant la journée ou la soirée d'hier.

(Lire la liste d'aliments. Surligner le type d'aliment consommé et cocher la colonne Oui ou Non si au

mois un aliment de la liste a été consommé ou non . Noter le nombre de fois par jour si nécessaire)

| *contenant de*  *la vitamine A* | Aliments à base d'huile de palme rouge, noix de palme  rouge ou sauce à base de noix de palme rouge |  |  |
| --- | --- | --- | --- |
| ***Groupe 7****:*  *Autres fruits*  *et légumes* | Tout autre fruit ou légume |  |  |
| ***Autres***  *(non*  *comptabilisé*  *dans le score*  *diététique)* | Tous huiles, gras, ou beurre ou aliments à base des  éléments ci-contre |  |  |
|  | Tous aliments sucrés, tels que chocolats, bonbons,  sucettes, pâtisseries, gâteaux ou biscuits |  |  |
|  | Condiments pour assaisonnement, tels que piments,  épices, herbes ou poudre de poisson |  |  |

**Q) K.1:** Concernant la femme en état de grossesse: Comment un femme enceinte doit-elle se nourrir en

comparaison avec une femme qui n'est pas en état de grossesse afin de fournir les nutriments nécessaires

à son bébé et contribuer à sa croissance ?

*Concernant la femme qui allaite: Comment une femme qui allaite doit-elle se nourrir en comparaison*

*avec une femme qui n'allaite pas afin d'être en bonne santé et de produire d'avantage de lait maternel?*

□ Manger d'avantage

□ Manger d'avantage à chaque repas ou fréquemment

□ Manger d'avantage d'aliments riches en protéines

□ Manger d'avantage d'aliments riches en fer

□ Cuisiner avec du sel iodisé

□ Autre( )

□ Je ne sais pas

**Q) K.2:** Certaines femmes consomment deux types de suppléments, ou tablettes, durant la grossesse.

Lesquels?

□ Suppléments de fer

□ Suppléments d'acide folique

□ Autre( )

□ Je ne sais pas

**Q) K.3:** Quels bénéfices résultent de la consommation de suppléments/tablettes d'acide folique?

□ Pour le développement normal du système nerveux du fœtus (cerveau, colonne vertébrale et

squelette)

□ Afin de prévenir des défauts/anomalies de naissance du système nerveux du fœtus (cerveau, colonne

vertébrale et squelette)

□ Autre( )

□ Je ne sais pas

**Q) K.4** Lorsqu'une femme en état de grossesse est mal nourrie, elle risque d'accoucher d'un bébé en

souspoids . Quels sont les risques sanitaires pour ces bébés?

□ Croissance et développement lents

□ Risques d'infections/de tomber malade

□ Risques de décès

□ Risques d'être mal nourri/d'avoir une déficience en micronutriments

□ Risques d'être malade à l'âge adulte/de développer une maladie chronique à l'âge adulte (maladie

cardiaque , tension élevée, obésité, diabètes)

□ Autre( )

□ Je ne sais pas

**Q) K.5:** Il est recommandé pour les femmes d'espacer leur grossesses de deux ou trois ans. Quelle est la

raison de cette recommandation?

□*Afin de reconstruire/recharger leurs réserves de nutriments (gras ,fer et autres)*

□*Afin que la mère puisse améliorer son état de santé avant d'avoir un autre bébé/afin de bien préparer*

*l'arrivée d'un nouveau bébé*

□ Autre( )

□*Je ne sais pas*

**Q) A. 1:** Quelle est, selon vous, la probabilité pour vous d'accoucher d'un bébé en souspoids?

□ Peut probable □ Je ne suis pas sûre □ Probable

*Si peut probable : Quelle en est la raison?*

_____________________________________________________

**Q) A. 2**: A quel point, selon vous, est-il grave d'accoucher d'un bébé en souspoids ?

□ Pas grave □ Je ne suis pas sûre □ Grave

*Si Pas grave :Quelle en est la raison?*

_____________________________________________________

**Q) A. 3 :** A quel point pensez-vous qu'il est bon de manger d'avantage durant la grossesse?

□ Pas favorable □ Je ne suis pas sûre □ Favorable

*Si Pas favorable: Quelle en est la raison?*

_____________________________________________________

**Q) A. 4 :** A quel point est-il difficile pour vous de manger d'avantage durant la grossesse?

□ Pas de difficulté □ Moyen □ Difficile

*Si Difficile: Quelle en est la raison?*

___________________________________________________
