# Supplementary material for: Factors influencing nutritional practices among mothers in Dakar, Senegal
Source: PLoS One. 2019 Feb 11;14(2):e0211787. doi: 10.1371/journal.pone.0211787 (PMC6370274; doi:10.1371/journal.pone.0211787)
Supplement: S2 File — (DOCX) [file pone.0211787.s002.docx]

**Agreement ver.4.0**

**Research theme**: Health and nutrition knowledge, attitudes and practices of mother and child in Dakar, Senegal.

1. I have read and understood the instructions provided above for this research and discussed with a researcher in charge about it.

2. I got a satisfactory answer about potential benefits and risks associated with the research.

3. I agree voluntarily to participate in this research.

4. I consent to the research staff collecting and processing my information within the purview permitted by related laws and IRB’s regulation.

5. I agree to an approved auditor appointed by the IRB, or any relevant regulatory authority or their approved representative reviewing my personal information for the sole purpose of checking the accuracy of the information recorded for the study.

6. I am aware that I have the right to withdraw from a study at any time and my decision to withdraw from this study doesn’t have any effect on my status.

7. My signature below indicates that I get a signed and dated copy of this form to keep, along with any other printed materials deemed necessary by the study investigators.

| Participant’s name |  | Signature |  | date |  |
| --- | --- | --- | --- | --- | --- |
| Researcher’ name |  | Signature |  | date |  |

**Instructions for respondents in research ver. 4.0**

**Research theme**: Health and nutrition knowledge, attitudes and practices of mother and child in Dakar, Senegal.

This research is to study on health and nutrition knowledge, attitude, and practice of pregnant and breast-feeding women. Before you agree to participate in this research study, you should carefully read the instruction and agreement. It is important that you understand research objective and process. The researcher in charge of this research, Professor Oh will explain about this research to you in detail. The research will be performed on only who voluntarily agree to participate in this research. Please take some time to read the following information and tell research team about your participation in the research. Also, you can discuss any issues related to this research with your family or friends if you wish. If you have any questions, research staff will explain the research study and answer any questions. Your signature on this form implies that you know and understand what the research is about and the risks and benefits of the research. Additionally, it means that you agree to participate in this research study.

1. Background and goal of the research

| - Every day, approximately 830 women after giving a birth die from preventable causes related to pregnancy and childbirth such as overbreeding, infection, high blood pressure, anemia, complications of labor and delivery, and so on. - The goal of research is to investigate on health and nutrition knowledge, attitudes, and practices of pregnant and breast-feeding women. |
| --- |

2. Research respondent

| - Pregnant or breast-feeding women and moms with children under 23 months of age are within the authority of three local health centers in Dakar area, Senegal. |
| --- |

3. Research Method

| - A paper survey |
| --- |

4. Survey Time

| - A survey that takes no more than 20 minutes to complete. |
| --- |

5. Benefit of taking part in research

| - The effects of indirect learning from survey questionnaire items. That is, respondent may gain a better understanding of his/her own health by answering survey. |
| --- |

6. Risk of taking part in research

| - This survey will take approximately 20 minutes. This may pose some inconvenience. Also, some questions are personal in nature. |
| --- |

7. Reward from participating research

| - Small gift for each respondent will be awarded |
| --- |

8. personal information and confidentiality

| - Answers from respondents will be used for only this research and be anonymous. All the collected data would be published and disposed after being kept hold in safe for 3 years; however, those can be opened for research verification by a reviewer or research committee that review research with the mandate according to research regulations if respondent’s privacy kept |
| --- |

9. Voluntarily research intonement and withdrawal

| - Subjects can choose whether to be this study or not. If respondent volunteers to be in this study, respondent may withdraw at any time without consequences of any kind. |
| --- |

10. Research inquiry

If you have any questions or concerns, please contact Oh, a research supervisor at 082)10-5088-7099 or katie5@snu.ac.kr. If you have any questions about your rights as a research subject, you may contact Institutional review board(IRB) managed by the Department of Health and Welfare of Korea at 082)2-737-8990 or katie5@snu.ac.kr.

File. Questionnaire

Characteristics

1. Age: ( )

2. Average gestational age: ( ) months

3. The number of children: ( )

4. Marital status

① Single   ② Married  ③ Divorced/separated

5. Household head

① Male headed   ② Female with male support   ③ Female without male support

6. Household head education level

① Less than primary   ② Primary school complete  ③ Above primary school

7. Mother’s education level

① Less than primary   ② Primary school complete   ③ Above primary school

8. Income generating activities

① Not involved   ② Involved in one activity ③ Involved in two activity ④ Involved in three activity

9. Does your household have a television?   □ Yes   □ No

10. Does your household have a refrigerator? □ Yes   □ No

11. Does your household have an MMDS/TV5 antenna? □ Yes   □ No

12. Does your household have a non-mobile telephone? □ Yes   □ No

13. Does your household have electricity? □ Yes   □ No

14. Does your household have a CD/DVD player? □ Yes   □ No

15. Does your household have an internet connection? □ Yes   □ No

16. Does your household have a CANAL television subscription? □ Yes   □ No

17. Does your household have a computer? □ Yes   □ No

18. Does any member of your household own their vehicle? □ Yes   □ No

19. Does any member of your household own their own cart? □ Yes   □ No

20. Does any member of this household have a bank account or account with another financial institution? □ Yes   □ No

21. What type of fuel does your household primarily use for cooking?

① Bottled gas   ② Wood, straw   ③ Other (                         )

22. What is the main source of drinking water for members of your household?

① Piped into dwelling   ② Unprotected wall   ③ Other (                         )

23. What kind of toilet facility do members of your household usually use?

① Flushed to piped sewer system   ② Flushed to septic tank   ③ Traditional latrine

④ Other (                         )

24. What is the primary material of the floor of your dwelling?

① Cement   ② Ceramic tile   ③ Earth/sand/dung   ④ Other (                         )

25. What is the primary material of the roof of your dwelling?

① Cement   ② Calamine/Cement fiber   ③ Other (                         )

26. What is the primary material used in the construction of the exterior walls of your dwelling?

① Cement   ② Bamboo/Cane/Palm/Trunks/Dirt   ③ Other (           )

Practices

Question P.1: Food-intake practices

I am going to ask you some questions about nutrition of pregnant and lactating women.

Q) P.1: Now I would like to ask you about liquids or foods that you ate yesterday during the day or at night.

(Read the food lists. Underline the corresponding foods consumed and tick the column Yes or No depending on whether any food item of the list was consumed. Record the number of times when relevant)

| *Group* | **Food lists** | **No** | **Yes** |
| --- | --- | --- | --- |
| ***Group 1****:  Grains, roots and tubers* | Porridge, bread, rice, noodles or other foods made from grains |  |  |
|  | White potatoes, white yams, manioc, cassava or any other foods made from roots |  |  |
| ***Group 2****: Legumes and nuts* | Any foods made from beans, peas, lentils, nuts or seeds |  |  |
| ***Group 3****:   Dairy products* | Milk, such as tinned, powdered or fresh animal milk |  | How many times? \|___\|\|___\| |
|  | Yogurt or drinking yogurt |  | How many times? \|___\|\|___\| |
|  | Cheese or other dairy products |  |  |
| ***Group 4****:   Flesh foods* | Liver, kidney, heart or other organ meats |  |  |
|  | Any meat, such as beef, pork, lamb, goat, chicken or duck |  |  |
|  | Fresh or dried fish, shellfish or seafood |  |  |
|  | Grubs, snails or insects |  |  |
| ***Group 5****:   Eggs* | Eggs |  |  |
| ***Group 6****:   Vitamin A fruits and vegetables* | Pumpkin, carrots, squash or sweet potatoes that are yellow or orange inside |  |  |
|  | Any dark green vegetables |  |  |
|  | Ripe mangoes (fresh or dried [not green]), ripe papayas (fresh or dried), musk melon |  |  |
|  | Foods made with red palm oil, red palm nut or red palm nut pulp sauce |  |  |
| ***Group 7****:   Other fruits and vegetables* | Any other fruits or vegetables |  |  |
| ***Others*** *(not counted in the dietary diversity score)* | Any oil, fats, or butter or foods made with any of these |  |  |
|  | Any sugary foods, such as chocolates, sweets, candies, pastries, cakes or biscuits |  |  |
|  | Condiments for flavour, such as chillies, spices, herbs or fish powder |  |  |

Knowledge

1 Question K.1: Women’s nutrition during pregnancy and breastfeeding

For a lactating woman:

How should a lactating woman eat in comparison with a non-lactating woman to be healthy and produce more breastmilk?

Please list four practices she should do.

____________________________________________________________________________

____________________________________________________________________________

____________________________________________________________________________

____________________________________________________________________________

- 1. Eat more food (more energy)
- Eat more at each meal (eat more food each day)

Or

- Eat more frequently (eat more times each day)
- 2. Eat more protein-rich foods
- 3. Eat more iron-rich foods
- 4. Use iodized salt when preparing meals
- Other
- Don’t know

Preliminary analysis

- Knows
- Does not know

Number of correct responses __

2 Question K.2: Micronutrient supplements for pregnant women

Most women would benefit from two types of supplements, or tablets, during pregnancy. Which are they?

____________________________________________________________________________

____________________________________________________________________________

____________________________________________________________________________

- Iron supplements
- Folic acid supplements
- Other
- Don’t know

Preliminary analysis

- Knows
- Does not know

2 Question K.3: Recommendation of folic acid supplements

Can you tell me why it is so important to take folic acid supplements during pregnancy?

Probe if necessary:

What is the health benefit for taking folic acid supplements/tablets?

____________________________________________________________________________

____________________________________________________________________________

____________________________________________________________________________

- For normal development of the nervous system of the unborn baby (brain, spine and skull)
- To prevent birth defects/abnormalities the nervous system of the unborn baby (brain, spine and skull)
- Other
- Don’t know

Preliminary analysis

- Knows
- Does not know

3 Question K.4: Health risks for low-birth-weight babies

When a pregnant woman is undernourished, she is at risk of having a low-birth-weight baby, meaning that the baby is small or has a low birth weight. What are the health risks for these babies?

____________________________________________________________________________

____________________________________________________________________________

- Slower growth and development
- Risks of infections/being sick
- Risks of dying
- Risks of being undernourished/having micronutrient deficiencies
- Risks of being sick once adult/developing chronic diseases in adulthood (heart disease, high blood pressure, obesity, diabetes)
- Other
- Don’t know

Preliminary analysis

Number of correct responses __

3 Question K.5: Family planning/birth spacing

**NOTE:** This question can generate anxiety in participants. The theme (family planning) should be handled with care.

It is recommended that a woman waits at least two or three years between pregnancies, that is before coming pregnant once again. Please can you tell me why this is recommended?

____________________________________________________________________________

____________________________________________________________________________

- To rebuild/fill up their body stores of nutrients (fat, iron and others)
- For the mother to be healthier before having a new baby/to be prepared for the arrival of a new baby⁭
- Other
- Don’t know

Preliminary analysis

- Knows
- Does not know

Attitudes

### Attitudes towards a health or nutrition-related problem

2 Giving birth/having a low-birth-weight baby

#### Perceived susceptibility

How likely do you think you are to have a low-birth-weight baby?

- 1. Not likely
- 2. You’re not sure
- 3. Likely

If Not likely:

Can you tell me the reason why it is not likely?

_____________________________________________________

_____________________________________________________

#### Perceived severity

How serious do you think it is for your baby to have a low-birth-weight?

- 1. Not serious
- 2. You’re not sure
- 3. Serious

If Not Serious:

Can you tell me the reason why it is not serious?

_____________________________________________________

_____________________________________________________

###

### Attitudes towards an ideal or desired nutrition-related practice

1 Eating more food during pregnancy: eating more at each meal or eating more frequently or having more snacks during the day

#### Perceived benefits

How good do you think it is to eat more food during pregnancy?

- 1. Not good
- 2. You’re not sure
- 3. Good

If Not good:

Can you tell me the reasons why it is not good?

_____________________________________________________

_____________________________________________________

#### Perceived barriers

How difficult is it for you to eat more food during pregnancy?

- 1. Not difficult
- 2. So-so
- 3. Difficult

If Difficult:

Can you tell me the reasons why it is difficult?

_____________________________________________________

_____________________________________________________
